# Blood Culture-Negative but Clinically Diagnosed Infective Endocarditis Complicated by Intracranial Mycotic Aneurysm, Brain Abscess, and Posterior Tibial Artery Pseudoaneurysm

**DOI:** 10.1155/2018/1236502

**Published:** 2018-11-08

**Authors:** Chao Jiang, Haibin Lu, Yaoqiang Guo, Li Zhu, Tianqi Luo, Wendy Ziai, Jian Wang

**Affiliations:** ^1^Department of Neurology, The Fifth Affiliated Hospital of Zhengzhou University, Zhengzhou 450052, China; ^2^Department of Neurology, Henan Provincial Chest Hospital, Zhengzhou 450000, China; ^3^Department of Anesthesiology/Critical Care Medicine, Johns Hopkins University, School of Medicine, Baltimore, MD, USA; ^4^Department of Neurology, Johns Hopkins University, School of Medicine, Baltimore, MD, USA

## Abstract

Blood culture-negative endocarditis is often severe and difficult to diagnose. It is necessary to emphasize the importance for the early diagnosis and accurate treatment of blood culture-negative endocarditis. Here, we described the relevant clinical information of a blood culture-negative but clinically diagnosed infective endocarditis complicated by intracranial mycotic aneurysm, brain abscess, and posterior tibial artery pseudoaneurysm. This patient was a 65-year-old man with a 9-month history of intermittent fever and died in the end for the progressive neurological deterioration. Although the blood culture is negative, this patient was clinically diagnosed as infective endocarditis according to Duke criteria. This patient course was complicated not only by cerebral embolism, intracranial mycotic aneurysm, and brain abscess but also by posterior tibial artery aneurysm of the lower extremity. The clinical findings of this patient suggest that the confirmatory microbiology is essential for the treatment of blood culture-negative infective endocarditis. Clinicians should be aware of the detriment of blood culture-negative infective endocarditis for its multiple complications may occur in one patient. The delayed etiological diagnosis and insufficient treatment may aggregate the clinical outcome of blood culture-negative infective endocarditis.

## 1. Introduction

Infective endocarditis (IE), with mortality rates of 20% to 40%, is the most frequently encountered endocarditis [1, 2]. However, many cases of IE are clinically silent and are recognized in only 2-10% of patients with eventual diagnosis of infective endocarditis [1, 2]. Neurological complications of IE, occurring in 20-40% of cases, can be classified into the following categories: meningitis-encephalopathy, ischemic complications, cerebral hemorrhage, and brain abscess [1]. Moreover, most complications occur in blood culture-positive infective endocarditis, and most patients only experience one or two neurological events during the IE episode [1]. Previous reports have indicated that blood culture-negative endocarditis is not common, and few studies have reported the correlation between blood culture-negative IE and neurological complications. Here, we report a blood culture-negative but clinically diagnosed patient with IE complicated by three neurological and one peripheral complication.

## 2. Case Report

**Figure 1:**
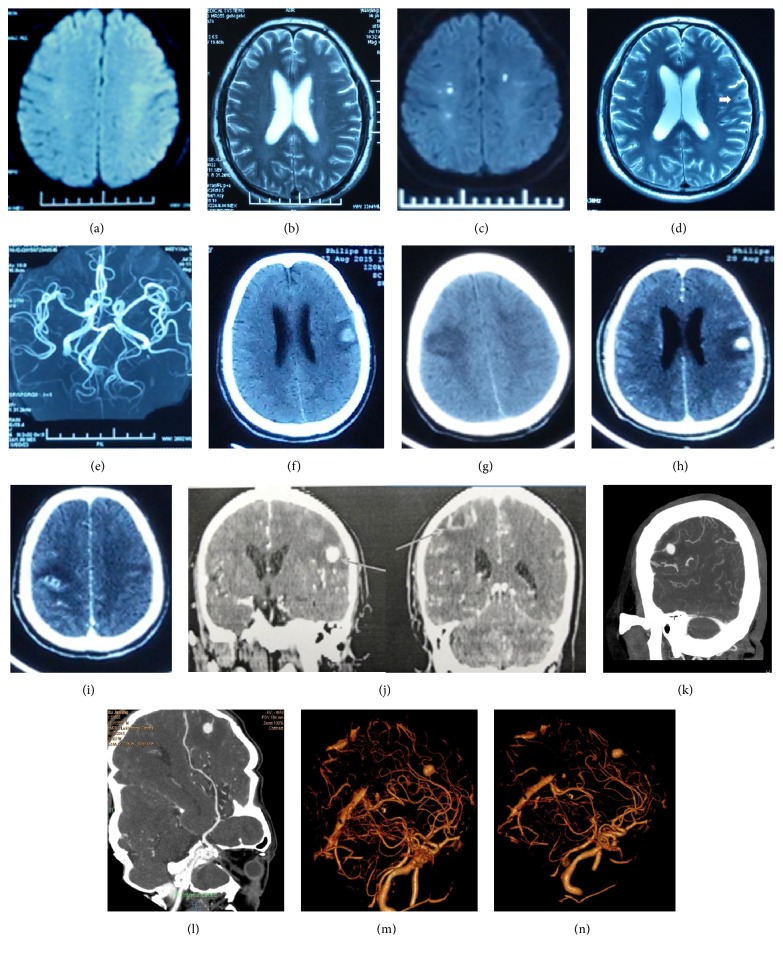
Dynamic changes on brain MRI and CT imaging. ((a) and (b)) On admission, DWI or T2 sequences of MRI showed hyperintense signal intensity in the right centrum semiovale and hyperintense lacunar lesions adjacent to the left lateral ventricle. ((c) and (d)) On day 7 of admission, MRI showed hyperintense signal intensity in the left cerebellar hemisphere, left temporal lobe, and bilateral frontal-parietal lobe. A flow void was found in the left hemisphere (arrow). (e) MRA did not show any abnormality in the intracranial arteries. ((f) and (g)) On day 11 of admission, CT scan revealed high density nodules in the right frontal-parietal region and a low density shadow in the right parietal lobe. ((h)–(j)) On day 20 of admission, Contrast-enhanced CT showed high density nodules surrounded by edema in the left frontal-parietal region and a ring-enhancing nodule in the right parietal lobe with peripheral edema (arrow). ((k)–(n)); CT angiography showed an aneurysm on a cortical branch of the left middle cerebral artery.

A 65-year-old man with a 9-month history of intermittent fever and pain in both lower extremities was admitted to our institution for distortion of commissure and numbness of the left upper limb. Physical examination revealed mild left facial paralysis, mild left hemiparesis, hypoalgesia of the left upper limb, and a fever of 38.5°C. Magnetic resonance imaging (MRI) of the head showed hyperintense signal intensity on diffusion weighted imaging or T2-imaging in the right centrum semiovale, and lacunar lesions in the left hemisphere (Figures 1(a) and 1(b)). A transesophageal echocardiogram revealed moderate regurgitation associated with large mobile vegetations on the aortic valves, measuring 14 by 6mm, mild regurgitation of mitral and tricuspid valves, enlargement of the left atrium, dilatation of the ascending aorta, and decrease in left ventricular diastolic function. Lab results were as follows: Anti-nuclear antibody (-), ANCA spectrum (-), Parasite antibody (-), T-spot (-), and Fungal D dextran assay (-). After treatment with aspirin and atorvastatin, the patient recovered quickly, and neurological symptoms resolved.

On day 7 of admission, the patient developed signs of unconsciousness and seizure like jerking in the limbs. Electroencephalogram (EEG) was normal after seizure like jerking. MRI of the head showed acute and subacute strokes in the brain (Figures 1(c) and 1(d)) and a flow void in the left hemisphere (Figure 1(d)). Magnetic Resonance Angiography (MRA) did not show any abnormalities in the intracranial arteries (Figure 1(e)). The computed tomography (CT) scan of chest, abdomen, and pelvis revealed that the solid tumor can be ruled out in this patient. Cerebral embolism was diagnosed as the most likely etiology of multifocal infarcts. Blood culture testing was conducted 3 times after admission but produced negative results. Infective endocarditis was diagnosed clinically according to the Duke criteria (one major and 3 minor) [1]. The major criteria for this patient were evidence of endocardial involvement with positive echocardiogram, and the minor criteria were the following: predisposing factor (prior antibiotic therapy), fever more than 38°C, and embolism evidence. This patient has received intermittent antibiotic therapy with amoxicillin and cephalosporins in the last 9 months. According to the principle of treatment for blood culture-negative endocarditis and the comparative efficacies of imipenem/cilastatin and vancomycin on ESBL producing Escherichia coli induced endocarditis [3, 4], the patient continued to receive antibiotic treatment with imipenem/cilastatin. It was also recommended to perform surgical treatment with aortic valve replacement as soon as possible. On day 11 of admission and before cardiac surgery, the patient developed signs of transient unconsciousness again. EEG was not performed immediately. With additional symptoms of aphasia and left limb weakness, a CT scan revealed high density nodules with surrounding edema in the right frontal-parietal region and a low density shadow in the right parietal lobe (Figures 1(f) and 1(g)). During this period, the patient's body temperature fluctuated between 36.5 and 38.4°C. We presumed the abnormal in CT image may be intracranial mycotic aneurysm and brain abscess according to the history and diagnosis of this patient.

On day 12 of admission, aphasia, left limb weakness, positive bilateral Chaddock sign, and a fever of 38.6°C were presented in this patient. In addition, a pulsatile mass was felt in the left posterior leg. Doppler ultrasound revealed a pseudoaneurysm measuring 32 by 30 mm in the left posterior tibial artery. Prolonged blood incubation was conducted another 3 times in this period, and the results remained negative. On day 20 of admission, the patient's condition suddenly deteriorated. He developed signs of confusion, and his gaze became fixed to the right side. Contrast-enhanced CT revealed high density nodules in the left frontal-parietal region and a ring-enhancing nodule in the right parietal lobe (Figures 1(h)–1(j)), suggesting the presence of a brain abscess. CT angiography showed an aneurysm, measuring 8 by 9mm of a cortical branch of the left middle cerebral artery (Figures 1(k)–1(n)). Then, the patient was discharged to home hospice care as family wishes. On follow-up, the family informed us that the patient had died at home one week after discharge.

## 3. Discussion

Blood culture-negative IE refers to endocarditis without etiology after three blood samples incubated on standard media [5]. The Duke criteria may be inadequate for blood culture-negative endocarditis due mainly to preceding antibiotic administration or to fastidious slow-growing organisms [6, 7]. Intracellular bacteria or autoimmunity also contributes to occurrence of culture-negative IE [7]. When autoimmune disorders and systemic cancer were ruled out, this patient who previously received intermittent antibiotic therapy was clinically diagnosed with blood culture-negative IE. This report suggests that even when blood cultures are negative on initial examination, careful history-taking, blood tests, and other auxiliary examination are crucial for diagnosis before histopathological examination. In addition, previous research has revealed the diagnostic value of PCR for blood culture-negative IE [5]. Although the positive rate of PCR for bacterial DNA is lower in blood samples than in valvular specimens in patients suspected to have IE [5], bacterial DNA was not sent due to the time limitation on the use of primers, which is a serious problem faced by clinicians before histopathological examination can be carried out.

Neurological complications are common manifestations of IE. They develop in 25% of cases and lead to a higher mortality rate, especially when vegetation size is ≥3 cm [8]. Septic embolization may cause ischemic stroke, mycotic aneurysm, intracranial hemorrhage, meningitis, and brain abscess [8, 9]. This patient manifested with multiple cerebral emboli, an intracranial mycotic aneurysm, a brain abscess, and a pseudoaneurysm in the left posterior tibial artery. Ischemic events are the most frequent neurologic complications of IE, accounting for about 42% of all neurologic complications, and are the first sign of IE in 47% of episodes [8]. Recurrent cerebral embolization has been rarely observed in previous reports [8]. We speculate this patient had recurrent cerebral embolization in a short period due to aortic valve vegetations. Although EEG is normal after the seizure like jerking, we also speculate that this patient may have had seizures contributing to transient encephalopathy for most of the EEG is negative in the internal of seizures [10]. Intracranial mycotic aneurysm is the consequence of displacement of septic emboli from valvular vegetations to the arterial vasa vasorum, disseminating the infection to the inner layer and wall of the vessel [11]. This complication is rare, occurring in 2-4% of cases [11, 12], and is typically located in the distal branches of the middle cerebral artery. The occurrence of brain abscess is also rare in patients with IE. One previous report revealed only 2 patients with brain abscesses in 208 cases of IE [1]. The presence of recurrent cerebral embolization, a mycotic aneurysm, and a brain abscess in a single patient has not been previously reported, which may most likely reflect the delayed diagnosis and inadequate use of antibiotics. This patient also had a pseudoaneurysm in the left posterior tibial artery. Previous reports have described the relationship between IE and the superior mesenteric artery or femoral pseudoaneurysm [13, 14]. However, no report has described posterior tibial artery pseudoaneurysm as a complication of IE.

In addition to the difficulty in diagnosis, the treatment for these complications does not yet have defined protocols, especially when occurring in combination. Early antimicrobial treatment and cardiac surgery can reduce the risk of cerebral embolism, intracranial mycotic aneurysm, brain abscess, and peripheral pseudoaneurysm [15]. Unruptured mycotic aneurysm may improve solely with antimicrobial treatment [2]. In addition, some research has suggested that, in the presence of mycotic aneurysms with cerebral infarction, the risk of rupture or hemorrhagic transformation warrants a 2-3 week postponement of cardiac surgery. With regard to the bacterial infection, the patient only received antimicrobial treatment with imipenem and cilastatin; infection associated with cocci, such as staphylococcus aureus and enterococcus, the most common causative organism, was not considered in the course of medical treatment.

Although previous report suggested that there was no difference in in-hospital mortality or long-term survival between culture-negative and culture-positive endocarditis patients [16, 17], the delayed etiological diagnosis may increase the mortality of culture-negative endocarditis patients. Absence of microbiological diagnosis may be a predictor for the poor prognosis of blood culture-negative endocarditis. Early diagnosis and empiric antibiotic treatment is important for the prognosis of blood culture-negative endocarditis.

## Abbreviations

IE: Infective endocarditis
MRI: Magnetic resonance imaging
MRA: Magnetic Resonance Angiography
EEG: Electroencephalogram
CT: Computed tomography.
